# Development of DArT (Diversity Arrays Technology) for high-throughput genotyping of *Pinus taeda* and closely related species

**DOI:** 10.1186/1753-6561-5-S7-P22

**Published:** 2011-09-13

**Authors:** Dione MT Alves-Freitas, Andrzej Kilian, Dario Grattapaglia

**Affiliations:** 1 EMBRAPA Genetic Resources and Biotechnology - EPqB Final W5 Norte 70770-917 Brazilia DF and Dep. Cell Biology, Universidade de Brazilia – UnB, Brazil; 2 Diversity Arrays Technology Pty Ltd, 1 Wilf Crane Crescent, Yarralumla, ACT 2600, Australia; 3 EMBRAPA Genetic Resources and Biotechnology - EPqB, 70770-910 Brazilia, Brazil; Dep. Cell Biology, Universidade de Brazilia – 70910-900 Brasília, Brazil.; Genomic Sciences Program - Universidade Católica de Brasília - Brazilia, Brazil

## Background

*Pinus *(*Pinaceae*) is the largest existing genus of conifers, with over 100 widely recognized species. Pines are an important source of wood, paper and resins, among others. Genetic improvement of *Pinus* species is a challenging endeavor. Breeding cycles typically last for decades, significant changes take place in wood properties and growth patterns with the transition from the juvenile to the adult phase and most traits are multi-factorial with low heritability [1]. Genomic Selection (GS) could radically reduce the time required for completion of a cycle of genetic improvement by precluding the progeny testing phase, significantly increasing the selection efficiency relative to conventional breeding [2,3]. To put GS into practice, genome-wide, high-throughput and cost-efficient marker are needed to advance this genomics assisted breeding approach focusing purely on prediction of performance, precluding gene-trait association discovery. To this end we are developing a high-throughput DNA genotyping platform for Pinus taeda based on the genome complexity reduction DArT (Diversity Arrays Technology) technology that has been successfully developed for many plant species including some with very large and complex genomes such as wheat [4]and sugarcane [5].

## Methods

For DArT array probe development population samples of 16 species of *Pinus*, 24 provenances of *Pinus taeda* L. and two mapping populations were used as starting material organized in pooled samples. For microsatellite and DArT mapping a set of 288 individually extracted haploid megagametophyte DNA samples of clone 7-56 were used. DArT development involved reduction of genome complexity of pooled samples by digestion with *PstI* as the primary cutter and *BstNI* as a frequently and secondary cutter followed byligation of adaptors to *PstI* overhangs and PCR amplification of intact *PstI* fragments following a protocol described earlier [6]. Genomic representations (targets) generated by PCR amplification were cloned into six separate libraries. A total of 7,680 clones were randomly picked being 2,304 clones from the 16 species libraries and 4,608 clones from *P. taeda* only libraries. A first test panel of 96 *Pinus* individuals was used to screen the 7,680 probes for polymorphic markers. Markers were declared reliable and polymorphic at a cutoff of 80% call rate, 95% reproducibility, and polymorphism information content > 0.1.Parallel to DArT marker development a linkage map of microsatellites was generated to provide a framework onto which the DArT markers will eventually be mapped. DNA from haploid megagametophyte samples of 288 seeds of Loblolly pine tree 7-56 was extracted. To immortalize the limited amount of haploid DNA of this mapping population, whole genome amplification was used. Haploid samples were genotyped with a selected set of 65 microsatellites. Genotyping was performed in an ABI PRISM 3100XL using 4-color fluorescent detection in multiplexed sets of 2 co-amplified microsatellites. Linkage analyses and map construction was carried out using JOINMAP 3.0.

## Results

A total of 856 and 930 markers were found polymorphic across samples of 24 individuals of each one of two mapping populations, while 1,776 DArT markers were polymorphic in a panel of 16 species, two individuals per species and 16 *Pinus taeda* US provenances, one individual per provenance. A significant number of probes could not be adequately scored due to background signal resulting from the presence of highly repetitive DNA that the *PstI/BstNI* complexity reduction could not remove from the 23 pg *Pinus taeda* genome. Cot-1 DNA, i.e. genomic DNA highly enriched for repetitive elements was therefore isolated from *P. taeda* DNA by shearing genomic DNA in autoclave, followed by DNA denaturing and re-annealing and S1 nuclease treatment. Using the Cot-1-DNA to quench repetitive probes, the recovery of assayable DArT markers was increased by 40%. When mapping microsatellites extensive locus duplication of the 65 microsatellite primer pairs resulted in a total of 182 individually "loci" scored for presence/absence in the haploid gametes. Based on the map position 96 of these "loci" actually corresponded to 48 effective loci and the other 86 are actual individual loci totaling 134. From the 134 segregating loci 110 were confidently mapped in the 12 expected linkage groups leaving two unlinked groups of 5 and 3 markers. Locus order was essentially the same for the 50 loci derived from 31 microsatellites in common with those recently mapped [7]. Interestingly, microsatellite PtTX3021 amplified 17 individually scored "loci". Eight were allelic pairs and were consolidated in four loci while the remaining 9 were unique. Additional duplications were seen for 15 microsatellites in different linkage groups suggesting paralogous loci.

## Conclusion

In spite of the complex and repetitive nature of the *Pinus taeda* genome several hundred DArT markers could be confidently genotyped indicating that DArT is a robust technology to be used for conifers. A new round of DArT probe picking and marker screening coupled to Cot-1 DNA use is now underway and should provide the target number of 7,680 selected probes for an operational array. The haploid mapping population will be genotyped with this array to provide information on distribution of the DArT markers in terms of recombination fraction and genome coverage relative to the framework of microsatellites. Additionally, probes detecting mapped markers will be sequenced to eventually aid the assembly of the 7-56 genome sequence.
